# Consumption

**Published:** 1855-10

**Authors:** 


### CONSUMPTION.

From official sources for the same year, it is found that of every hundred persons dying from all causes, there died in round numbers, of consumption, in

| | Consumption. | Of all lung diseases. |
|---|---|---|
| Mexico City, | 3 | 17 |
| New Orleans, | 9 | 14 |
| Norfolk, | 11 | 13 |
| Philadelphia, | 15 | 29 |
| Boston, | 15 | 24 |
| New York, | 18 | 28 |
| Baltimore, | 18 | 23 |
| Charleston, | 18 | 23 |
| Havana, | 20 | 25 |

As far as it is safe to form an opinion from official statistical returns, every family in the Union which has the slightest possible consumptive taint ought to vote for the annexation of Mexico, *instanter*, *vi et armis vel*, hook, crook, or money; and by a parity of reasoning, we don't want Cuba as a gift, for already consumption destroys a sixth of our entire population. Cuba would increase that mortality over twenty per cent.

